# Supplementary material for: A theoretical model of factors influencing online consumer purchasing behavior through electronic word of mouth data mining and analysis
Source: PLoS One. 2023 May 18;18(5):e0286034. doi: 10.1371/journal.pone.0286034 (PMC10194948; doi:10.1371/journal.pone.0286034)
Supplement: Attachment — Submitted filename: Response to Reviewers.docx [file pone.0286034.s002.docx]

Dear Editors and Reviews:

Thank you for your correspondence and for the feedback provided by the reviewers regarding our manuscript titled “A Theoretical Model of Factors Influencing Online Consumer Purchasing Behavior through Electronic Word of Mouth Data Mining and Analysis”. These valuable and constructive comments have assisted in revising and enhancing our paper, in addition to providing significant guidance to our research. We have carefully examined and addressed all provided comments, and hope that the corrections meet with approval. The main revisions made to the paper and our corresponding responses to the reviewers' comments are listed below.

Responds to the Journal Requirements:

1. Please ensure that your manuscript meets PLOS ONE's style requirements, including those for file naming.

Response: We have modified our manuscript to ensure compliance with PLOS ONE's style requirements, including those for file naming.

2. Please state what role the funders took in the study. If the funders had no role, please state: "The funders had no role in study design, data collection and analysis, decision to publish, or preparation of the manuscript."

If this statement is not correct you must amend it as needed.

Please include this amended Role of Funder statement in your cover letter; we will change the online submission form on your behalf.

Response: The cover letter contains a statement indicating that "The funders had no role in study design, data collection and analysis, decision to publish, or preparation of the manuscript."

3. In your Data Availability statement, you have not specified where the minimal data set underlying the results described in your manuscript can be found. PLOS defines a study's minimal data set as the underlying data used to reach the conclusions drawn in the manuscript and any additional data required to replicate the reported study findings in their entirety. All PLOS journals require that the minimal data set be made fully available.

Response: We have uploaded a separate file consisting of the study's minimal data.

4. PLOS requires an ORCID iD for the corresponding author in Editorial Manager on papers submitted after December 6th, 2016. Please ensure that you have an ORCID iD and that it is validated in Editorial Manager.

Response: The corresponding author has updated the ORCID iD.

5. Please ensure that you include a title page within your main document. You should list all authors and all affiliations as per our author instructions and clearly indicate the corresponding author.

Response: we have included a title page with information for all authors in our main document.

6. Please review your reference list to ensure that it is complete and correct. If you have cited papers that have been retracted, please include the rationale for doing so in the manuscript text, or remove these references and replace them with relevant current references.

Response: Our reference list has been carefully reviewed and verified to be both complete and accurate.

Special thanks to you for your good comments.

Responds to the reviewers’ comments:

Reviewer #1: As a journal reviewer, I would like to commend the authors for their rigorous data analysis process and the comprehensive results presented in the article. However, there are two minor aspects that need to be addressed by the authors in order to increase the quality of this paper.

The introduction section can be strengthening by clearly highlighting the research problem, significance, and gaps in the literature. This would help outline the main contributions of this submission.

It has been noticed that you have cited a bit old research (2010, 2011, 2013). You are suggested to update these citations with recent and well-established ones.

Response: Firstly, the introduction section has been rewritten to address the reviewers' comments. The research problem, significance, and gaps in the literature have all been included in the introduction. Secondly, outdated references from 2010, 2011, and 2013 have been removed and replaced with more recent references.

Special thanks to you for your good comments.

Reviewer #2: This is a well-written paper with good contributions. All important elements of a good academic research paper are presented in this submission. I would suggest a few minor amendments.

1. The abstract appears to be more focused on reporting results rather than effectively communicating the central idea(s) explored in the paper and the contribution it makes to the scientific community's existing knowledge. Its lack of coherence makes it challenging to follow.

Response: The abstract section has been rewritten to enhance the logical flow and coherence of the paper, as suggested by the reviewers.

2. The discussion section should first compare the findings of this research with those of prior research. Both theoretical and practical implications should be reported in a separate section.

Response: In the discussion section, we have compared the findings of our research with those of prior research, and have separated the theoretical and practical implications into distinct sections.

3. The literature review, particularly the literature related to the SOR and WOM, should be strengthen by including well-established and related research.

This includes but is not limited to:

- E-commerce in high uncertainty avoidance cultures: The driving forces of repurchase and word-of-mouth intentions. Doi: https://doi.org/10.1016/j.techsoc.2022.102083

- Boosting Online Purchase Intention in High-Uncertainty-Avoidance Societies: A Signaling Theory Approach. Doi: https://doi.org/10.3390/joitmc8030136

- Solving the product uncertainty hurdle in social commerce: The mediating role of seller uncertainty. Doi: https://doi.org/10.1016/j.jjimei.2023.100169

- Boosting Customer E-Loyalty: An Extended Scale of Online Service Quality. Doi:https://doi.org/10.3390/info10120380

- Building customer loyalty in online shopping: the role of online trust, online satisfaction and electronic word of mouth. Doi: <https://doi.org/10.1504/IJEMR.2020.108132>

Response: Additionally, we have incorporated the listed references suggested by the reviewer to strengthen the literature review.

Special thanks to you for your good comments.

We have made our best effort to improve the manuscript and greatly appreciate the Editors and Reviewers' earnest work. We hope that the revised manuscript meets with approval.

Thank you once again for your valuable comments and suggestions.

Best regards,

Dr. Xiaoya Zhu
